# A Case Report of Giant Bilateral Wolffian Adnexal Tumor

**DOI:** 10.1002/cnr2.70084

**Published:** 2024-12-18

**Authors:** Ling Huang, Yan Zhou, Xiaoshan Hong, Xiping Luo, Min Shen, Shanshan Yan, Xiaoli Sun

**Affiliations:** ^1^ Department of Gynaecology Guangdong Women and Children Hospital Guangzhou China; ^2^ Department of Radiology Guangdong Women and Children Hospital Guangzhou China; ^3^ Department of Pathology Guangdong Women and Children Hospital Guangzhou China

**Keywords:** bilateral, MRI, pericardial effusion, pleural effusion, Wolffian tumor

## Abstract

**Background:**

Wolffian adnexal tumor is a rare type of tumor that was first discovered and reported by Karim‐inejad in 1973. Wolffian adnexal tumor lacks specific clinical manifestations and its histological morphology is similar to various other tumors, making it highly prone to misdiagnosis. To enhance our understanding of this disease, we hereby report a case of Wolffian adnexal tumor diagnosed and treated in our hospital.

**Case:**

It is the first report of a giant bilateral Wolffian tumor, with pleural effusion and pericardial effusion as the initial symptoms. Magnetic resonance imaging (MRI) suggested a huge lobulated mass (25.3 × 17.8 × 21.9 cm) in the mid‐lower abdomen and pelvis, involving both ovaries. A diagnosis of “ovarian malignancy” was made before the surgery. Hysterectomy, bilateral adnexectomy, and omentectomy were performed. Postoperative pathology revealed a bilateral Wolffian tumor. Postoperative chemotherapy with a taxol and cisplatin (TP) regimen was administered for six cycles. Follow‐up at 2 months postoperatively showed resolution of pericardial and pleural effusions, and there has been no recurrence during the 3‐year follow‐up period.

**Conclusion:**

Wolffian adnexal tumor lacks specific clinical manifestations, and its prognosis is good after treatment.

## 1 Introduction

Wolffian adnexal tumor is a rare type of tumor that was first discovered and reported by Karim‐inejad in 1973 [1]. So far, approximately 130 cases have been reported in English literature. Wolffian tumor mainly occurs in the areas where the remnants of the mesonephric duct are distributed, and it is most commonly found in the broad ligament, mesosalpinx, and ovarian hilum. Wolffian adnexal tumor lacks specific clinical manifestations and its histological morphology is similar to various other tumors, making it highly prone to misdiagnosis [2, 3].

To enhance our understanding of this disease, we hereby report a case of Wolffian adnexal tumor diagnosed and treated in our hospital. Currently reported Wolffian tumors mainly affect one side of the adnexa, but this case presents the first report of a huge bilateral Wolffian tumor, with pleural effusion and pericardial effusion as the initial symptoms. The immunohistochemical profile of Wolffian tumors is nonspecific, and the MRI features of Wolffian tumors are rarely reported. This case report also provides a detailed record of the MRI characteristics of bilateral adnexal Wolffian tumors in the patient, which can serve as a valuable reference for future clinical diagnosis and treatment.

## 2 Case

The patient, a 58‐year‐old female, was admitted to the Guangdong Women and Children Hospital on January 21, 2021, due to postmenopausal vaginal bleeding for over a year and abdominal distension for half a year. Two months ago, the patient was hospitalized in the respiratory department of another hospital due to coughing and shortness of breath and was considered to have pleural effusion. After thoracentesis, her symptoms were relieved, and no tumor cells were found in the pleural effusion examination. Abdominal ultrasonography revealed a huge pelvic and abdominal mass with unclear boundaries. The previous hospital suspected Megis syndrome and recommended further treatment. Since the onset of the illness, the patient's bowel and urination functions have been normal, and there has been no significant weight loss. For the past 2 months, she has been unable to lie flat to sleep, but her daily activities such as walking and household chores have not been affected. She has a history of hypertension for over 10 years and gout for over 2 years, without regular treatment. She has had four pregnancies, with one live birth and three abortions.

Physical examination revealed edema in both lower limbs with varicose veins, abdominal distension, prominent umbilicus, and a palpable round, firm mass in the abdomen with clear boundaries, extending from below the xiphoid process to above the symphysis pubis, and laterally to the anterior axillary lines on both sides. The mass had good mobility, no tenderness, and no rebound tenderness. The cervix was smooth, and uterine palpation was unclear.

**FIGURE 1:**
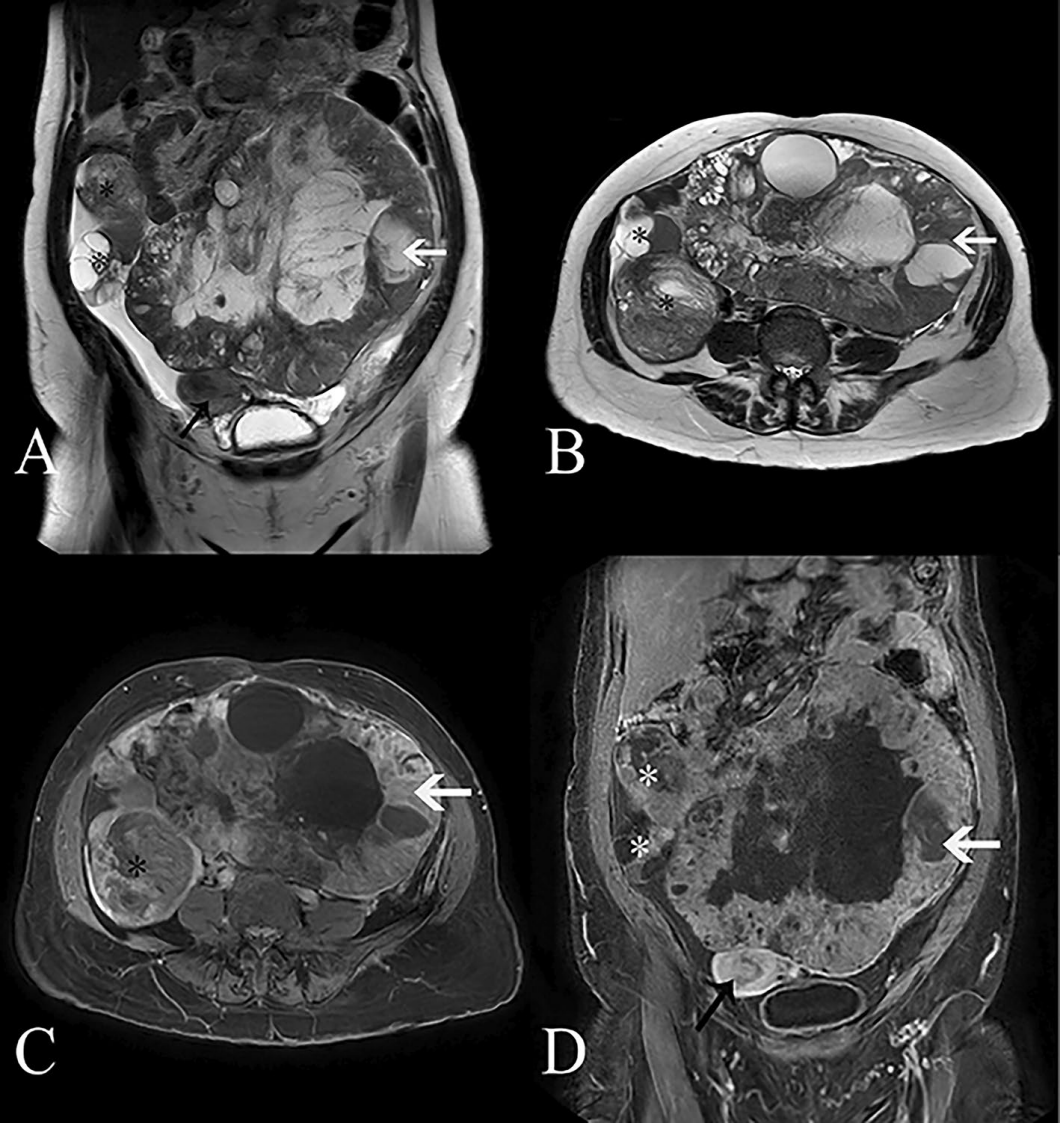
Image of MRI (A) on the coronal T2‐weighted sequence, the left ovarian tumor (indicated by the white arrow) exhibits cystic and solid changes with mixed high and low T2 signals. Similarly, the right ovarian tumor (indicated by *) also displays cystic and solid changes with mixed high and low T2 signals. The uterus (indicated by the black arrow) is compressed and shifted toward the lower right. (B) Axial T2‐weighted sequence shows cystic and solid changes in the left ovarian tumor (indicated by the white arrow), with mixed high and low T2 signals. Similarly, the right ovarian tumor (*) also exhibits cystic and solid changes with mixed high and low T2 signals. (C) Coronal T1‐weighted enhanced sequence demonstrates significant and inhomogeneous enhancement in the solid portion of the left ovarian tumor (indicated by the white arrow), while the cystic portion shows no significant enhancement. The solid part of the right ovarian tumor (*) also exhibits significant and inhomogeneous enhancement, with no apparent enhancement in the cystic portion. (D) Axial T1‐weighted enhanced sequence reveals significant and inhomogeneous enhancement in the solid part of the left ovarian tumor (indicated by the white arrow), while the cystic part does not show significant enhancement. Similarly, the solid portion of the right ovarian tumor (*) demonstrates significant and inhomogeneous enhancement, with no observable enhancement in the cystic part. The uterine muscle layer enhances uniformly.

**FIGURE 2:**
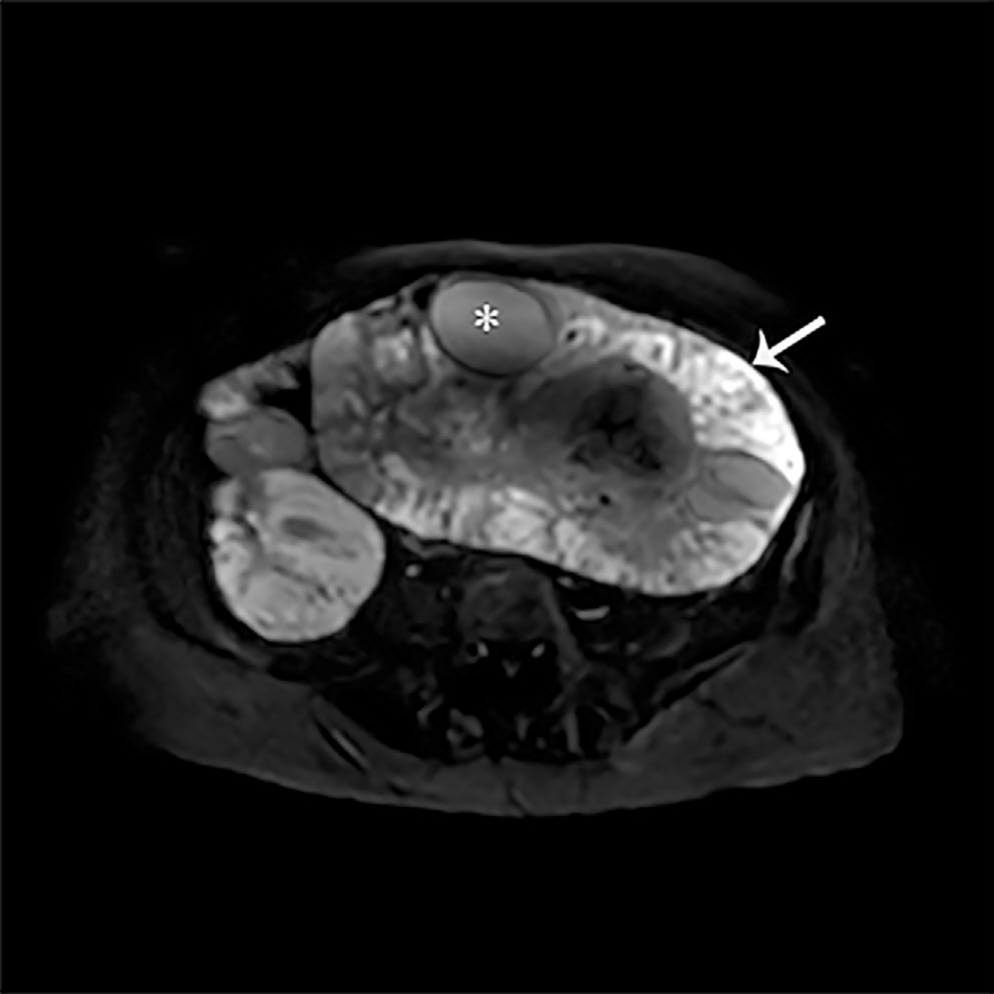
Image of MRI on diffusion‐weighted imaging (DWI), the densely populated areas of tumor cells (indicated by the white arrow) exhibit high‐signal intensity, while the cystic and necrotic regions of the tumor (indicated by *) show low signal intensity.

Auxiliary examinations included ultrasonography, which revealed pericardial effusion and right pleural effusion. MRI imaging suggested a huge lobulated mass (25.3 × 17.8 × 21.9 cm) in the mid‐lower abdomen and pelvis, involving both ovaries, with a high probability of malignancy originating from the left adnexa (Figures 1 and 2). Laboratory tests showed elevated levels of CA125, CA199, and HE4. Hormone levels were within normal ranges.

A diagnosis of “ovarian malignancy” was made, and laparotomy was performed on January 27, 2021. Intraoperatively, a huge pelvic and abdominal mass approximately 25 × 25 × 20 cm in size was found, mainly solid, with a yellowish‐white and smooth surface originating from the left ovary. The mass was widely adherent to the intestines, resulting in fixation, and the mesenteric vessels were rich and dilated. After resection of the left adnexa (weighing 3.27 kg), a right adnexal mass measuring 8 × 10 × 10 cm was exposed, also mainly solid, with a smooth capsule, no adhesions to the surrounding tissues, and abundant, dilated, and tree‐like vessels on the surface. No normal ovarian tissue was observed, and the fallopian tube was attached to the enlarged ovary. The omentum was slightly pale without obvious nodules, and there were scattered reddish flocculent tissues measuring 0.5–0.8 cm on the surface of the small and large intestines. Subsequently, hysterectomy, bilateral adnexectomy, and omentectomy were performed. The cut surface of the left adnexal mass was mainly solid with partial cystic areas and a yellowish color. Intraoperative bleeding was 3000 mL due to extensive intestinal adhesions and rich vasculature.

**FIGURE 3:**
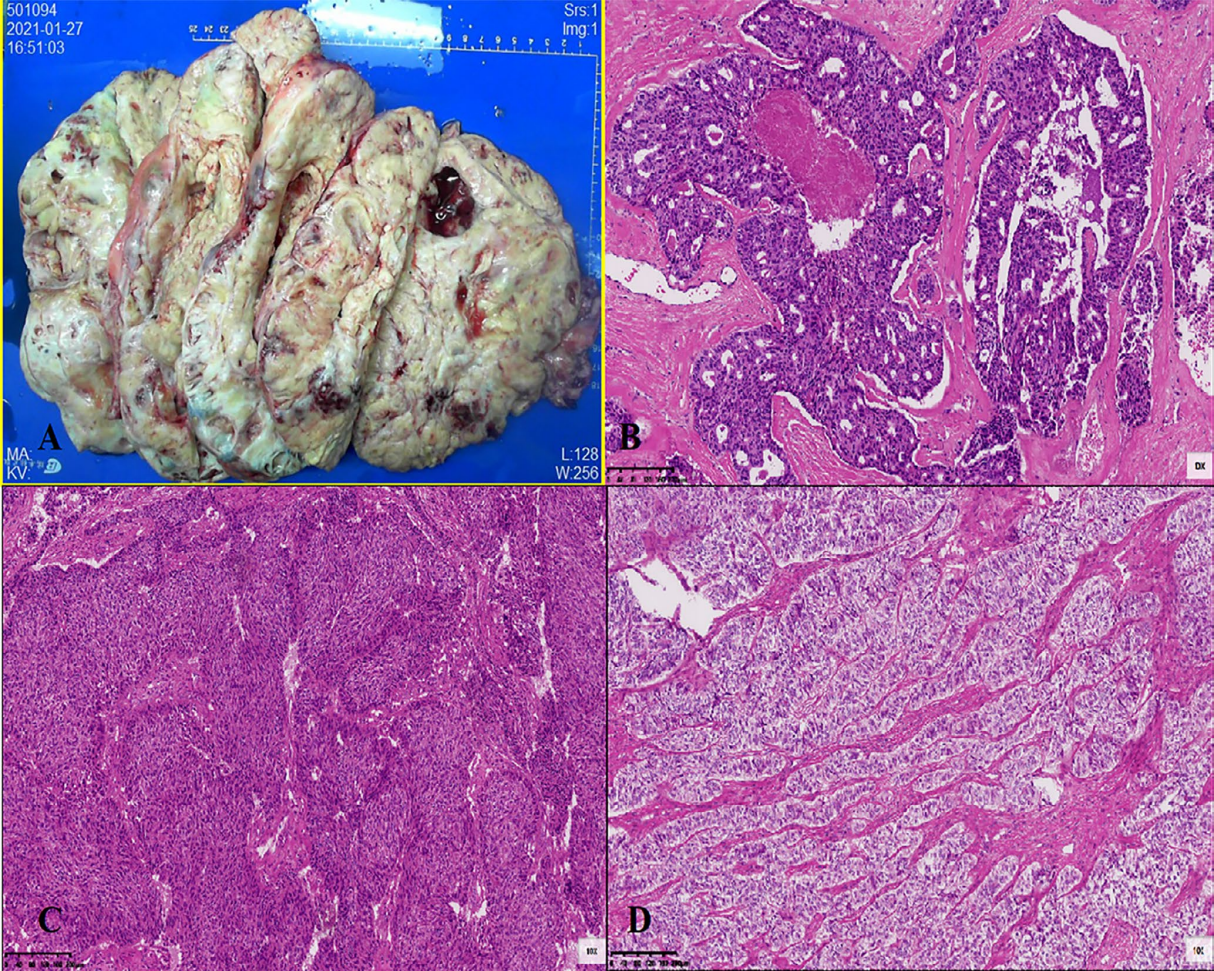
Pathological tumor features (A) gross features: A large, irregular tumor with a grayish‐white solid cut surface accompanied by cystic changes and necrosis. (B–D) Hematoxylin–eosin (original magnification× 100): Microscopically, the tumor cells are arranged in solid sheets, sieve‐like patterns, glands, papillae, trabeculae, and adenoma‐like structures. Locally, the cytoplasm appears transparent with mucinous stroma. The nuclei are moderately atypical, and the number of mitoses is less than 5 per 10 high‐power fields (HPF).

**FIGURE 4:**
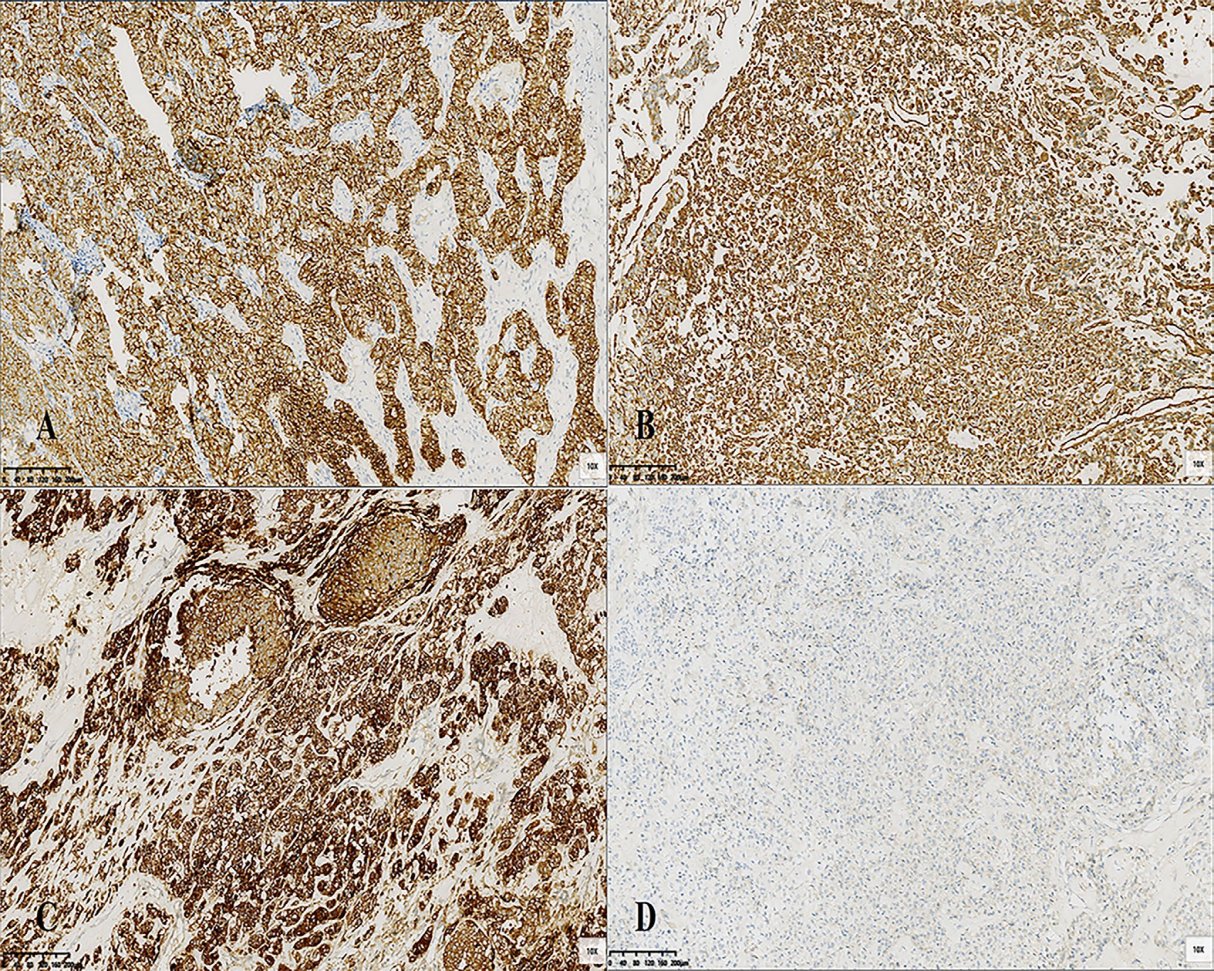
Representative images of immunohistochemical stains. (A) Positive immunostaining for CK‐pan. (B) Positive immunostaining for Vimentin. (C) Positive immunostaining for CD10. (D) Negative immunostaining for a‐inhibin (original magnification× 100 [A–D]).

Postoperative pathology revealed a Wolffian tumor involving the left ovary, right fallopian tube, and ovary (Figure 3). The left fallopian tube, omentum, and mesenteric surfaces were not involved. The endometrium showed cystic atrophic changes. Immunohistochemical staining of the tumor tissue showed positive staining for CK‐pan, partial positivity for CK7, negativity for EMA, positivity for Vimentin, negativity for PAX‐8, positivity for ER in 80% of cells, positivity for PR in 10% of cells, negativity for α‐inhibin, Calretinin, focal positivity for Syn, positivity for CD10, negativity for GATA3, TTF‐1, partial positivity for CD99, negativity for p53, and focal weak positivity for WT‐1. The Ki‐67 proliferation index was 20% (Figure 4).

The final diagnosis was mesonephric duct adnexal tumor (stage IIA). The levels of CA199 and HE4 returned to normal after operation, and the level of CA125 descended after surgery. The postoperative chemotherapy with a TP regimen was administered for six cycles. The levels of tumor marker returned to normal after six cycles of chemotherapy. Follow‐up at 2 months postoperatively showed resolution of pericardial and pleural effusions, and there has been no recurrence during the 3‐year follow‐up period.

### 2.1 Ethical Approval and Informed Consent

The institutional review board of Guangdong Women and Children Hospital approved this case report. The written informed consent to publish her case including images was given by the patient.

## 3 Discussion

Wolffian tumors, also known as mesonephric tumors, are classified as low‐grade malignancies according to the 2014 WHO classification [4]. Previous literature reports indicate that the onset age ranges from 15 to 83 years, with a median age of 50 years. Most cases are incidentally discovered (53%), and symptomatic patients often present with abdominal pain, abdominal distension, vaginal bleeding, etc. The average size of the tumor is 6.1 cm (ranging from 0.8 to 25.0 cm), and it is typically unilateral, making preoperative diagnosis difficult. Imaging examinations reveal that the tumor envelope is thick and shows a homogeneously hypoechoic pattern on ultrasound. CT typically demonstrates a multiloculated mass with clear boundaries, and irregular enhancement of the lesion envelope suggests malignancy. MR imaging shows an oval‐shaped mass with clear boundaries. On T2‐weighted images, the tumor appears as a high‐signal mass with cystic components in the center. On T1‐weighted images with contrast enhancement, the mass demonstrates uniform enhancement, while the cystic portion remains unenhanced. During surgery, the tumor is observed to originate from residual Wolffian duct regions such as the ovarian hilum, mesosalpinx, parauterine region, and even the paravaginal area. Grossly, the tumor appears solid, oval, rounded, nodular, or lobulated, with a smooth and shiny envelope. It is yellowish or light gray in color, and occasionally there may be focal hemorrhage with central cystic necrosis [3]. No specific tumor marker changes are observed in this type of tumor, although occasional cases of elevated CA125 have been reported [5]. Pathologically, there are no specific immunohistochemical markers, leading to diagnostic difficulties [6].

The clinical presentation and imaging findings of this case are consistent with previous literature reports, but the presence of a huge mass involving both adnexa is relatively rare. Despite the large size of the tumor and its involvement in the abdominal cavity, there is no evidence of intraperitoneal or distant metastasis. Therefore, the patient does not exhibit symptoms of cachexia, and the long duration of the disease reflects the low‐grade malignant nature of the Wolffian tumor, which is consistent with the preoperative assessment of the tumor's pathological type.

It is worth noting that the patient presented with a pelvic‐abdominal mass accompanied by pericardial effusion and pleural effusion, which led to a diagnosis of Megis syndrome at an external hospital. Megis syndrome refers to the occurrence of ascites and pleural effusion in patients with benign solid ovarian tumors, which resolve rapidly after tumor resection. It was first reported by Megis et al. in 1937. The tumor types associated with Megis syndrome include ovarian fibroids, ovarian thecoma, and granulosa cell tumors. Subsequently, literature reports have described other pathological types of pelvic benign and malignant tumors, such as ovarian benign teratomas, uterine leiomyomas, and papillary tumors of the fallopian tube, that can be associated with ascites and pleural effusion, termed pseudo‐Megis syndrome [7]. However, both Megis syndrome and pseudo‐Megis syndrome involve the simultaneous occurrence of ascites and pleural effusion. In this case, there were no ascites; therefore, a diagnosis of Megis syndrome or pseudo‐Megis syndrome cannot be made. The cause of the pleural effusion and pericardial effusion remains unclear. Based on the clinical presentation (pleural effusion, pericardial effusion, and edema of lower extremities), hypoproteinemia is the initial consideration, but it is difficult to explain the absence of ascites.

Furthermore, the surgical procedure in this case was challenging and risky. Although the tumor did not invade abdominal organs, the intestines were adherent to the mass, and the mesenteric vessels were dilated. After separation, the mesenteric vessels ruptured, resulting in extensive bleeding and transient shock. Fortunately, due to adequate preoperative preparation, the situation was stabilized.

Although Wolffian tumors are considered low‐grade malignancies, they still have the potential for recurrence and metastasis. The rate of metastasis and recurrence is approximately 11%, and the earliest recurrence can occur within 2 years of diagnosis. Common sites of metastasis are the liver and lungs. The average time to recurrence is 48 months, and most recurrences occur in cases where only the tumor was resected [8, 9]. Due to the limited number of cases, no clinical or pathological indicators associated with prognosis have been observed, and there is no standardized postoperative treatment protocol. Some literature suggests that tumor necrosis, capsular invasion, high mitotic count, positive CD117, and overexpression of Ki‐67 may indicate malignancy [10]. In this case, the tumor is classified as stage IIA and is relatively large. Therefore, postoperative chemotherapy is recommended based on the treatment of malignant epithelial ovarian tumors, and long‐term follow‐up is necessary to monitor the prognosis. Encountering a huge pelvic mass, Wolffian adnexal tumor is a rare but possible cause. Although wolffian adnexal tumor is a type of low‐grade malignant tumor, it still has the possibility of recurrence and metastasis. Radical operation and necessary adjuvant chemotherapy may be beneficial in reducing recurrence and metastasis.

Several case reports about female adnexal tumors of probable Wolfan origin have been published previously [11, 12]. The latest case report by Filippo Alberto Ferrari et al. reported that the tumor markers were normal, and the patient was asymptomatic at the time of the diagnosis. The patient underwent surgery without any adjuvant therapy postoperatively, and there has been no recurrence during follow‐up [11]. In this case report, the clinical symptoms of the patient differ from those we reported. Despite the absence of adjuvant therapy during the treatment process, the patient has had a good prognosis, which also indicates the nonspecificity of the clinical symptoms of this tumor and its relatively favorable prognosis.

## 4 Conclusion

In summary, Giant bilateral wolffian adnexal tumor is an rare disease. This disease exhibits various morphological patterns, and there is no consistent standard for its treatment. This case report provides a detailed record of the MRI characteristics and treatment procedure of bilateral adnexal Wolffian tumors in the patient, which can serve as a valuable reference for future clinical diagnosis and treatment.

## Author Contributions

**Ling Huang:** investigation, writing – original draft, writing – review and editing. **Yan Zhou:** writing – review and editing. **Xiaoshan Hong:** writing – review and editing. **Xiping Luo:** writing – review and editing. **Min Shen:** writing – review and editing, investigation. **Shanshan Yan:** investigation. **Xiaoli Sun:** conceptualization, investigation, writing – review and editing, writing – original draft.

## Conflicts of Interest

The authors declare no conflicts of interest.

## Data Availability

All data generated or analyzed during this study are included in the article. Further inquiries can be directed to the corresponding author.
